# Supplementary material for: Essential function of the alveolin network in the subpellicular microtubules and conoid assembly in Toxoplasma gondii
Source: eLife. 2020 May 7;9:e56635. doi: 10.7554/eLife.56635 (PMC7228768; doi:10.7554/eLife.56635)
Supplement: Supplementary file 3. [file elife-56635-supp3.docx]

| **Key Resources Table** | | | | | |
| --- | --- | --- | --- | --- | --- |
| **Reagent type (species) or resource** | **Designation** | **Source or reference** | **Identifiers** | **Additional information** |  |
| strain, strain background  (*T. gondii*) | *T. gondii*: Strain RH/ΔHX/ΔKU80 | ATCC | Ref# 50174 |  |  |
| strain, strain background  (*T. gondii*) | AC9-mAID-HA | This study |  | Generated in a Tir1 expressing strain using CRISPR/Cas9 |  |
| strain, strain background  (*T. gondii*) | AC10-mAID-HA | This study |  | Generated in a Tir1 expressing strain using CRISPR/Cas9 |  |
| strain, strain background  (*T. gondii*) | AC9-mAID-HA / Cen2-Ty | This study |  | Knock-in generated in the AC9-mAiD-HA strain using CRISPR/Cas9 |  |
| strain, strain background  (*T. gondii*) | AC9-mAID-HA / RNG2-Ty | This study |  | Knock-in generated in the AC9-mAiD-HA strain using CRISPR/Cas9 |  |
| strain, strain background  (*T. gondii*) | AC9-mAID-HA / CPH1-Ty | This study |  | Knock-in generated in the AC9-mAiD-HA strain using CRISPR/Cas9 |  |
| strain, strain background  (*T. gondii*) | AC9-mAID-HA /GAP70mycGFP | This study |  | Second copy expressed in the AC9-mAiD-HA strain |  |
| strain, strain background  (*T. gondii*) | AC9-mAID-HA / AC10-Ty | This study |  | Knock-in generated in the AC9-mAiD-HA strain using CRISPR/Cas9 |  |
| strain, strain background  (*T. gondii*) | AC9-mycBirA | This study |  | Generated in an RH strain using CRISPR/Cas9 |  |
| strain, strain background  (*T. gondii*) | AC10-mAID-HA / AC9-Ty | This study |  | Knock-in generated in the AC10-mAiD-HA strain using CRISPR/Cas9 |  |
| strain, strain background  (*T. gondii*) | AC9-mAID-HA / AC2-Ty | This study |  | Knock-in generated in the AC10-mAiD-HA strain using CRISPR/Cas9 |  |
| strain, strain background  (*T. gondii*) | AC9-mAID-HA / AC8-Ty | This study |  | Knock-in generated in the AC10-mAiD-HA strain using CRISPR/Cas9 |  |
| strain, strain background  (*T. gondii*) | AC10-mAID-HA / AC2-Ty | This study |  | Knock-in generated in the AC10-mAiD-HA strain using CRISPR/Cas9 |  |
| strain, strain background  (*T. gondii*) | AC10-mAID-HA / AC8-Ty | This study |  | Knock-in generated in the AC10-mAiD-HA strain using CRISPR/Cas9 |  |
| strain, strain background  (*T. gondii*) | AC10-mAID-HA / RNG2-Ty | This study |  | Knock-in generated in the AC10-mAiD-HA strain using CRISPR/Cas9 |  |
| strain, strain background  (*T. gondii*) | AC2-mAID-HA | This study |  | Generated in a Tir1 expressing strain using CRISPR/Cas9 |  |
| strain, strain background  (*T. gondii*) | AC8-mAID-HA | This study |  | Generated in a Tir1 expressing strain using CRISPR/Cas9 |  |
| strain, strain background  (*T. gondii*) | AC9-mAID-HA / MyoH-Ty | This study |  | Knock-in generated in the AC9-mAiD-HA strain using CRISPR/Cas9 |  |
| strain, strain background  (*T. gondii*) | AC10-mAID-HA / MyoH-Ty | This study |  | Knock-in generated in the AC10-mAiD-HA strain using CRISPR/Cas9 |  |
| strain, strain background  (*T. gondii*) | AC9-mAID-HA / AKMT-Ty | This study |  | Knock-in generated in the AC9-mAiD-HA strain using CRISPR/Cas9 |  |
| strain, strain background  (*T. gondii*) | AC9-mAID-HA / FRM1-Ty | This study |  | Knock-in generated in the AC9-mAiD-HA strain using CRISPR/Cas9 |  |
| strain, strain background  (*T. gondii*) | AC9-mAID-HA / ICMAP1-Ty | This study |  | Knock-in generated in the AC9-mAiD-HA strain using CRISPR/Cas9 |  |
| strain, strain background  (*T. gondii*) | AC9-mAID-HA / APR1-Ty | This study |  | Knock-in generated in the AC9-mAiD-HA strain using CRISPR/Cas9 |  |
| strain, strain background  (*T. gondii*) | AC10-mAID-HA / KinA-Ty | This study |  | Knock-in generated in the AC10-mAiD-HA strain using CRISPR/Cas9 |  |
| strain, strain background  (*T. gondii*) | AC9-mAID-HA / RNG1-Ty | This study |  | Knock-in generated in the AC9-mAiD-HA strain using CRISPR/Cas9 |  |
| strain, strain background  (*T. gondii*) | AC10-mAID-HA / RNG1-Ty | This study |  | Knock-in generated in the AC10-mAiD-HA strain using CRISPR/Cas9 |  |
| cell line (*Homo sapiens*) | Human Foreskin Fibroblasts (HFFs) | ATCC | Ref# CCD1072Sk |  |  |
| Antibody | Anti-GAP45 (Rabbit polyclonal) | **Plattner *et al.,* 2008**  DOI: 10.1016/j.chom.2008.01.001 |  | IF (1:1000) |  |
| Antibody | Anti-IMC1 (Rabbit polyclonal) | **Frénal *et al.,* 2014**  DOI: 10.1371/journal.ppat.1004504 |  | IF (1:1000) |  |
| Antibody | Anti-ARO (Rabbit polyclonal) | **Mueller *et al.,* 2013**  DOI: 10.1016/j.chom.2013.02.001 |  | IF (1:1000) |  |
| Antibody | Anti-GAC (Rabbit polyclonal) | **Tosetti *et al.,* 2019**  DOI: 10.7554/eLife.42669 |  | IF (1:3000) |  |
| Antibody | Anti-polyglutamate chain (Poly-E)  (Rabbit polyclonal) | AdipoGen | Ref# AG-25B-0030 | IF (1:500) |  |
| Antibody | Anti-HA (Rabbit polyclonal) | Sigma | Ref# H6908 | IF (1:1000)  WB (1:1000) |  |
| Antibody | Anti-ACT (Mouse monoclonal) | **Herm-Götz *et al.,* 2002**  DOI: 10.1093/emboj/21.9.2149 |  | WB (1:1000) |  |
| Antibody | Anti-ISP1 (Mouse monoclonal) | **Beck *et al.,* 2010**  DOI: 10.1371/journal.ppat.1001094 |  | IF (1:3000) |  |
| Antibody | Anti-SAG1 (Mouse monoclonal) | Other |  | Clone T4-1E5(Hybrydoma supernatant) IF (1:10) |  |
| Antibody | Anti-Ty (Mouse monoclonal) | Other |  | Clone BB2  (Hybrydoma supernatant) IF (1:10) |  |
| Antibody | Anti-ROP2-4 (Mouse monoclonal) | Other |  | Gift from J-F Dubremetz  IF (1:1000) |  |
| Antibody | Anti-alpha tubulin (Guinea Pig monoclonal) | **Le Guennec *et al.,* 2020**  DOI: 10.1126/sciadv.aaz4137 | Ref# AA345 scFv-F2C | IF (1:250) |  |
| Antibody | Anti-beta tubulin (Guinea Pig monoclonal) | **Le Guennec *et al.,* 2020**  DOI: 10.1126/sciadv.aaz4137 | Ref# AA344 scFv-S11B | IF (1:250) |  |
| Antibody | Anti-acetylated α-tubulin (Mouse monoclonal) | Santa Cruz Biotechnology | Ref# sc-23950 (6-11 B-1) | IF (1:1000) |  |
| Antibody | Alexa Fluor 405 - Anti-mouse (Goat polyclonal) | Invitrogen | Ref# A31553 | IF (1:3000) |  |
| Antibody | Alexa Fluor 488 - Anti-mouse (Goat polyclonal) | Invitrogen | Ref# A11001 | IF (1:3000) |  |
| Antibody | Alexa Fluor 568 - Anti-mouse (Goat polyclonal) | Invitrogen | Ref# A11004 | IF (1:3000) |  |
| Antibody | Alexa Fluor 594 - Anti-mouse (Goat polyclonal) | Invitrogen | Ref# A11005 | IF (1:3000) |  |
| Antibody | Alexa Fluor 405 - Anti-rabbit (Goat polyclonal) | Invitrogen | Ref# A31556 | IF (1:3000) |  |
| Antibody | Alexa Fluor 488 - Anti-rabbit (Goat polyclonal) | Invitrogen | Ref# A11008 | IF (1:3000) |  |
| Antibody | Alexa Fluor 568 - Anti-rabbit (Goat polyclonal) | Invitrogen | Ref# A11011 | IF (1:3000) |  |
| Antibody | Alexa Fluor 594 - Anti-rabbit (Goat polyclonal) | Invitrogen | Ref# A11012 | IF (1:3000) |  |
| Antibody | Alexa Fluor 568 - Anti-guinea pig (Goat polyclonal) | Invitrogen | Ref# A11075 | IF (1:400) |  |
| Antibody | Alexa Fluor 594 - Anti-guinea pig (Goat polyclonal) | Invitrogen | Ref# A11076 | IF (1:400) |  |
| Antibody | Anti-rabbit IgG - HRP (Goat polyclonal) | Sigma | Ref# A8275-1ML | WB (1:1000) |  |
| Antibody | Anti-mouse IgG - HRP  (Goat polyclonal) | Sigma | Ref# A5278-1ML | WB (1:1000) |  |
| recombinant DNA reagent | pTUB1:YFP-mAID-3HA, DHFR-TS:HXGPRT | Addgene | RRID: Addgene_87259 | Template for mAiD-HA PCRs (KI) |  |
| recombinant DNA reagent | ASP5-3Ty-D  HFR | **Hammoudi *et al.,* 2015**  DOI: 10.1371/journal.ppat.1005211 |  | Template for 3Ty PCRs (KI) |  |
| recombinant DNA reagent | MycBirA | **Long *et al.,* 2018**  DOI: 10.21769/BioProtoc.2768 |  | Template for MycBirA PCRs (KI) |  |
| recombinant DNA reagent | pSAG1::Cas9-U6::sgUPRT | Addgene | RRID: Addgene_54467 | Backbone for gRNA |  |
| sequenced-based reagent | AC9 gRNA | This paper | PCR primers | GAACATAGATAGAAAATCTGGTTTTAGAGCTAGAAATAGC |  |
| sequenced-based reagent | AC10 gRNA | This paper | PCR primers | GTTGCTCTGGCAGGAGATTGGTTTTAGAGCTAGAAATAGC |  |
| sequenced-based reagent | AC2 gRNA | This paper | PCR primers | GCAGTTGTGGAACCCATAGACGTTTTAGAGCTAGAAATAGC |  |
| sequenced-based reagent | AC8 gRNA | This paper | PCR primers | GAGAAAGGAAGAAACGCACAGTTTTAGAGCTAGAAATAGC |  |
| sequenced-based reagent | Centrin2 gRNA | This paper | PCR primers | GGAAGCAGGGACGTTTCACAGTTTTAGAGCTAGAAATAGC |  |
| sequenced-based reagent | MyoH gRNA | This paper | PCR primers | GCAACTGACGCAAGACTTGCGGTTTTAGAGCTAGAAATAGC |  |
| sequenced-based reagent | RNG1 gRNA | This paper | PCR primers | GGTGTCGGCGATTTGGCGAAGTTTTAGAGCTAGAAATAGC |  |
| sequenced-based reagent | KinA gRNA | This paper | PCR primers | ATCTGGAGCGAAGCGTTCAAGTTTTAGAGCTAGAAATAGC |  |
| commercial assay or kit | Q5® Site-Directed Mutagenesis Kit | New England Biolabs | Ref# E0554S |  |  |
| commercial assay or kit | KOD | Novagen | Ref# 71085-3 |  |  |
| commercial assay or kit | Microtubule Binding Protein Spin-Down Assay Biochem Kit | Cytoskeleton, Inc. | Ref# BK029 |  |  |
| chemical compound, drug | Biotin | Sigma-Aldrich | Ref# 47868 |  |  |
| chemical compound, drug | 3-indole acetic acid (IAA) | Sigma-Aldrich | Ref# I2886 |  |  |
| chemical compound, drug | Pyrimethamine | Sigma-Aldrich | Ref# P7771 | 1 µg/ml |  |
| chemical compound, drug | Mycophenolic acid | Sigma-Aldrich | Ref# M5255 | 25 mg/mL |  |
| chemical compound, drug | Xanthine | Sigma-Aldrich | Ref# X0626 | 50 mg/mL |  |
| chemical compound, drug | BIPPO; (5-Benzyl-3-isopropyl-1H-pyrazolo[4,3-d]pyrimidin-7(6H)-one) | **Howard *et al.,* 2015**  DOI: 10.1021/cb501004q |  |  |  |
| chemical compound, drug | Calcium Ionophore A23187 | Sigma-Aldrich | Ref# C7522 |  |  |
| chemical compound, drug | Deoxycholate | Sigma-Aldrich | Ref# D6750 |  |  |
| software, algorithm | ImageJ | NIH | RRID: SCR_003070 | https://imagej.  nih.gov/ij/ |  |
| software, algorithm | GraphPad Prism 8 | GraphPad | RRID: SCR_002798 | https://www.graphpad.  com/scientific-  software/prism/ |  |
